# Data for the physical and mechanical properties of staple fibers cement paste composites

**DOI:** 10.1016/j.dib.2017.07.055

**Published:** 2017-07-27

**Authors:** Ertug Aydin

**Affiliations:** Department of Civil Engineering, European University of Lefke, Lefke, Mersin 10, North Cyprus, Turkey

## Abstract

The data presented herein are compiled of the research summary of “Staple-wire-reinforced high-volume fly-ash cement paste composites” (Aydin, in preparation) [1]. This data article provides general information about the novel high volume fly ash cement paste composites composed of various volume of staple wires. The dataset here also helps the readers to understand the mechanisms of staple wires on physical and mechanical properties of pure cement paste composites.

**Specifications Table**

**Table:** 

Subject area	*Civil Engineering, Material Science Engineering*
More specific subject area	*Physical and mechanical properties*
Type of data	*Images, Figures, Text File*
How data was acquired	*Physical and mechanical tests (Laboratory), Equations [2,3]*
Data format	*Raw, Analyzed*
Experimental factors	*The six different volume fraction of staple wire fiber, high volume fly ash and cement are used to manufacture the cement paste composites in a small mold.*
Experimental features	*Various volume of staple wire fibers are blended with high volume fly ash cement paste composites to investigates the physical and mechanical properties.*
Data source location	*Mersin 10 Turkey, Lefke, North Cyprus*
Data accessibility	*The all data herein and supplementary files are all available within this article.*
Related research article	E. Aydin, Staple wire-reinforced high-volume fly-ash cement paste composites, Constr. Build. Mater., 2017 (in preparation).

**Value of the data**

• The data presented herein can be used to investigate the effects of different length of staple fiber.
• The dataset can be used by others to investigate further properties of staple wire fiber.
• The data presented herein may be used to develop new methods by using different fibers.
• The research data may be helpful for manufacturing commercially sustainable building products.

## 1 Data

The dataset presented herein were obtained from the physical and mechanical tests for various volume proportions of staple wire fiber blended with high volume fly ash (HVFA) and cement. The data provides in this article composed of pure cement paste composites. The detailed of the dataset presented here can be found in [1]. Additionally, the existing models proposed by others [2], [3] were used to check the applicability for staple wire HVFA cement paste composites. The regression analysis of test data for 336 samples were used to predict physico-mechanical properties of the staple wire-reinforced paste composites.

## 2 Experimental design, materials and methods

**Fig. 1:**
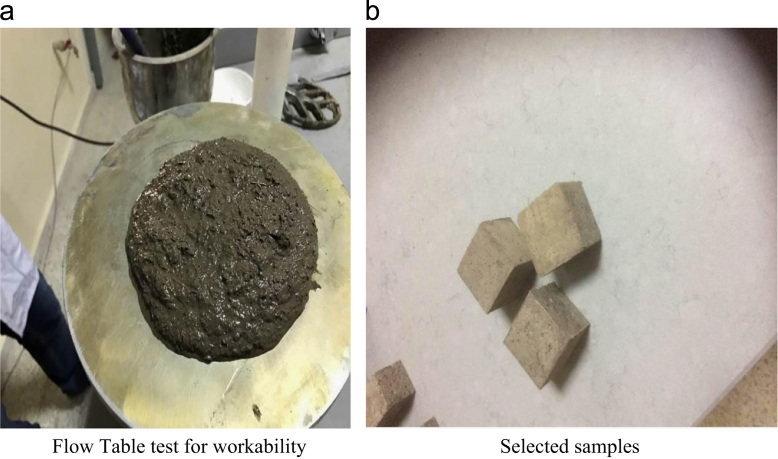
**a.** Flow Table test for workability. **b.** Selected samples.

**Fig. 2:**
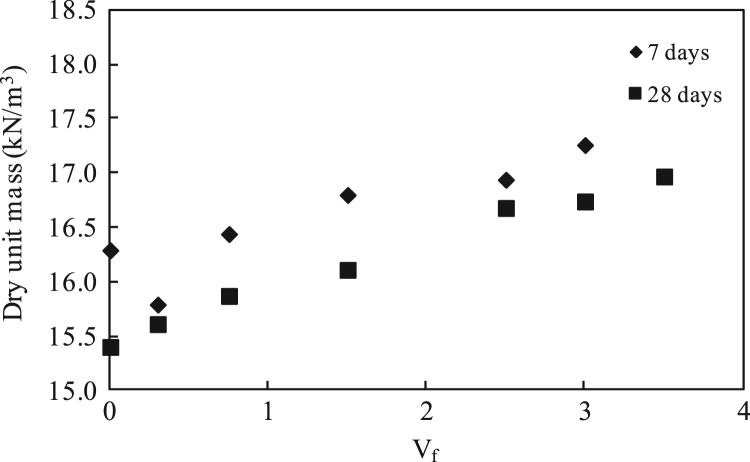
Dry unit mass versus V_f_ for high-volume fly-ash staple fiber composites at 7-and 28-days.

**Fig. 3:**
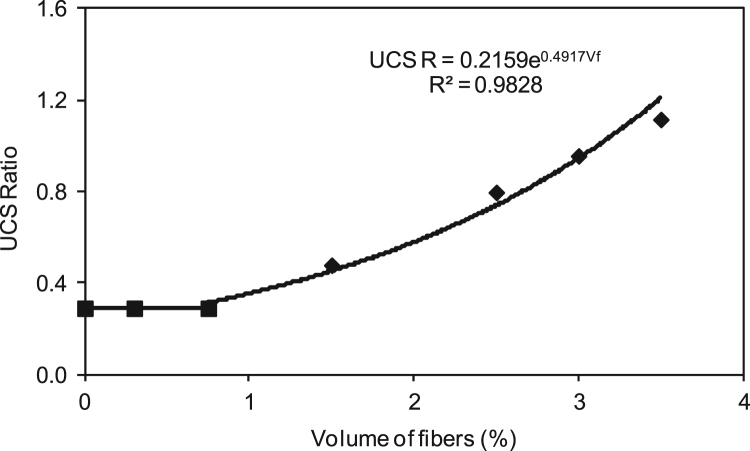
Correlation of unconfined compressive strength Ratio versus volume of staple wire fiber.

**Fig. 4:**
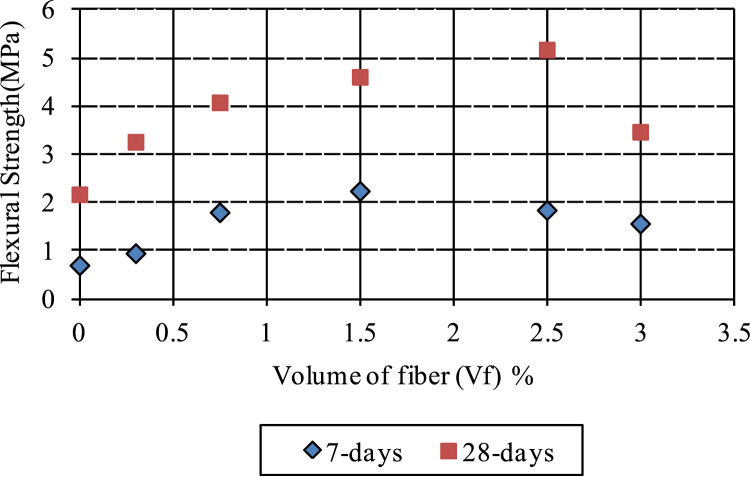
Flexural strength versus volume of staple fiber at 7 and 28 days.

**Fig. 5:**
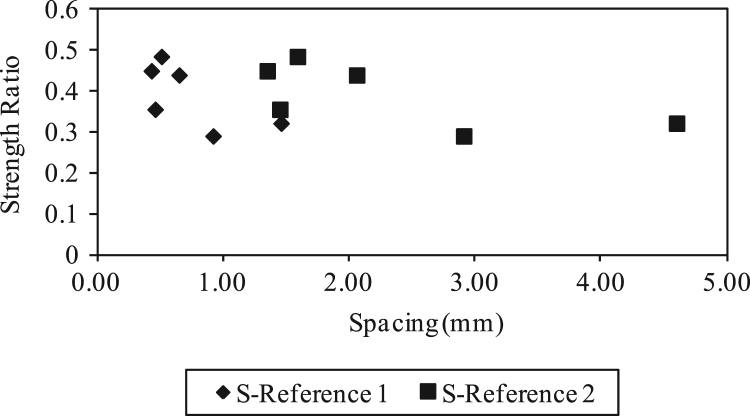
Strength ratio versus spacing of staple wire fiber cement paste composites according to reference 1 [2], [3], [9] and reference 2 [2], [3], [10].

**Fig. 6:**
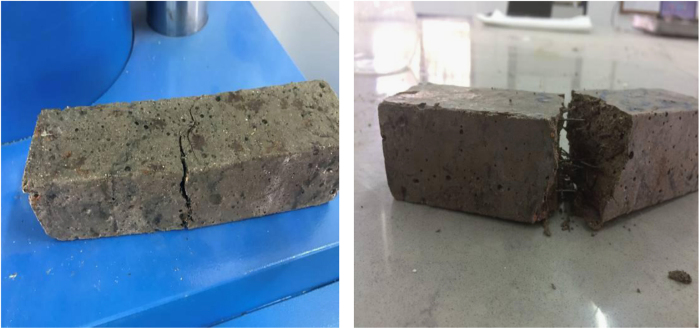
Cracked samples after flexural strength test.

**Fig. 7:**
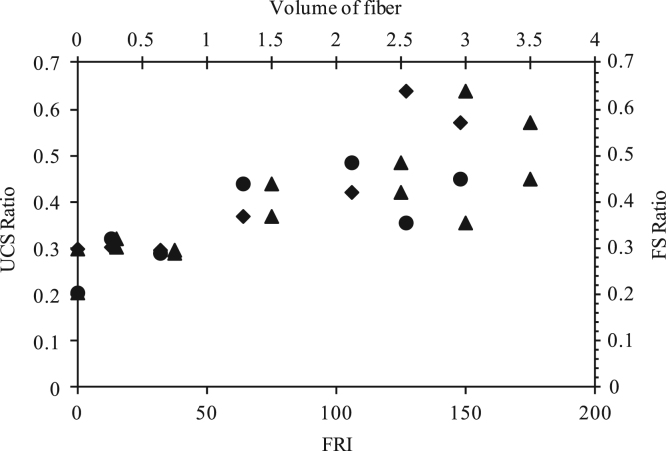
Unconfined compressive strength (UCS) ratio and flexural strength (FS) ratio versus Fiber reinforcement index (FRI) [*V*_f_*l/*d, l denotes length, d denotes diameter*] and volume of fiber.

**Fig. 8:**
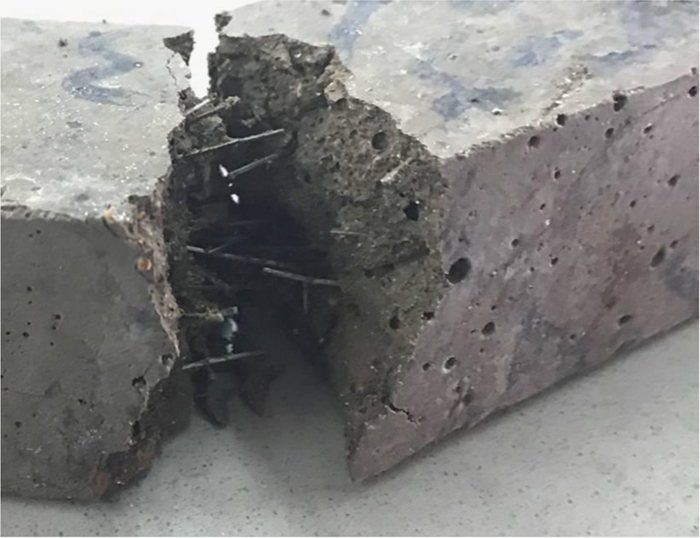
Staple wire fibers (dispersion) after flexural strength test.

**Fig. 9:**
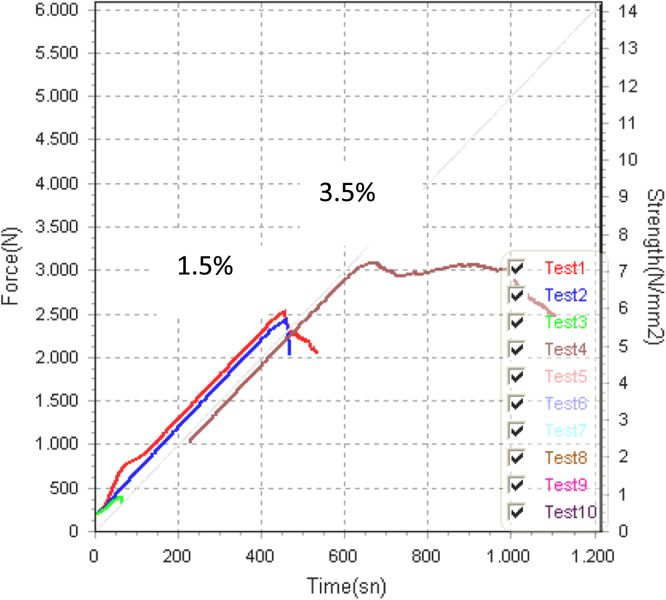
Force (N)/Strength (MPa) versus Time (s) graphs for composites composed 3.5% and 1.5% volume of staple fiber.

The water-to-cement (w/c) ratio was kept constant at 39.5% for all mixtures, as optimized in a previous research [4], [5]. The data presented here examined 80% fly ash, 20% cement, and staple fiber ranging from 0% to 3.5% by volume of paste. Different HVFA cement paste mixes were experimentally examined, and mixes which showed the best performance were chosen for the previous research [4], [5]. Composites were cast in 50 mm cubic molds and 40 mm×40 mm×160 mm prismatic molds. Previous equations for spacing proposed by other researchers [2], [3] have been used to check their validity of volume of staple wire fiber of high volume fly ash cement paste composites. Additionally, based on the ACI report [6] and ASTM standards [7], [8], alternative areas of application of this composite in construction section were investigated. The detailed of mix proportions, experimental setup and results can be found in [1] (Fig. 1, Fig. 2, Fig. 3, Fig. 4, Fig. 5, Fig. 6, Fig. 7, Fig. 8, Fig. 9)
