# Supplementary material for: Data for the physical and mechanical properties of staple fibers cement paste composites
Source: Data Brief. 2017 Jul 27;14:307–12. doi: 10.1016/j.dib.2017.07.055 (PMC5544468; doi:10.1016/j.dib.2017.07.055)
Supplement: Supplementary file 1 — Supplementary material [file mmc1.docx]

There are no conflicts of interest to declare
